# Construction and effect evaluation of perioperative nursing model for gastric cancer patients based on enhanced recovery after surgery (ERAS) concept

**DOI:** 10.1097/MD.0000000000046009

**Published:** 2025-12-12

**Authors:** Yu Song, Yang Zhao, Rui Wang, Ting Wu, Xinhua Liao

**Affiliations:** a General Surgery Department, The First Affiliated Hospital of Xi’an Jiaotong Universiy, Xi’an, Shaanxi, China.

**Keywords:** enhanced recovery after surgery (ERAS) concept, gastric cancer, patient satisfaction, perioperative nursing, postoperative complications, postoperative recovery

## Abstract

This study explores the application effect of the nursing model based on the concept of enhanced recovery after surgery (ERAS) in the perioperative period of gastric cancer patients, aiming to optimize the nursing process, accelerate postoperative recovery, and improve the quality of nursing care. A retrospective analysis was conducted on 120 patients who underwent radical gastrectomy for gastric cancer in our hospital from January 2022 to December 2024. The patients were divided into an observation group and a control group, with 60 cases in each group. The control group received the traditional perioperative nursing model, while the observation group received the nursing model based on the ERAS concept. The 2 groups were compared in terms of postoperative hospital stay, incidence of complications, time to first flatus, time to first out-of-bed activity, patient satisfaction, pain score, and psychological score. The observation group had a significantly shorter postoperative hospital stay than the control group (7.2 ± 1.5 days vs 10.5 ± 2.1 days, *P* < .001). The total incidence of complications was significantly lower in the observation group than in the control group (10.0% vs 26.7%, *P* = .013). The time to first flatus (24.5 ± 5.2 hours vs 36.8 ± 6.5 hours, *P* < .001) and the time to first out-of-bed activity (20.3 ± 4.1 hours vs 30.5 ± 5.3 hours, *P* < .001) were both significantly shorter in the observation group than in the control group. The patient satisfaction score was significantly higher in the observation group than in the control group (92.5 ± 5.6 points vs 80.2 ± 6.8 points, *P* < .001). The postoperative pain score was significantly lower in the observation group than in the control group, and the psychological score was significantly better in the observation group than in the control group. The nursing model based on the ERAS concept can significantly shorten the postoperative hospital stay of gastric cancer patients, reduce the incidence of postoperative complications, promote the recovery of postoperative gastrointestinal function and the improvement of early mobilization ability, and at the same time, improve patient satisfaction, reduce postoperative pain, and improve psychological status. It has important clinical application value and is worthy of promotion and application in clinical practice.

## 1. Introduction

Gastric cancer is one of the most common malignant tumors worldwide, with high incidence and mortality rates that pose a serious threat to human health.^[1,2]^ According to the World Health Organization, gastric cancer ranks among the top causes of cancer-related deaths globally.^[3,4]^ Its high incidence and mortality are related to the fact that early symptoms of gastric cancer are not obvious and most patients are diagnosed at an advanced stage. Surgical resection is the primary treatment for gastric cancer, especially for patients with early-stage and some mid-stage gastric cancer, as it can effectively extend the patients’ survival period.^[5]^ However, the traditional perioperative nursing model often has many shortcomings, such as long postoperative recovery time, high complication rate, and increased hospitalization costs, which bring a heavy burden to patients and their families.

In recent years, with the shift in medical paradigms and the continuous updating of nursing concepts, the enhanced recovery after surgery (ERAS) concept has gradually emerged and been widely applied in clinical practice.^[6,7]^ ERAS is a multimodal perioperative management strategy based on evidence-based medicine, aiming to optimize every link in the perioperative period, reduce surgical stress responses, accelerate postoperative recovery, improve the quality of life for patients, and reduce medical costs. The core content of this concept includes optimized preoperative preparation, minimally invasive surgery, and early postoperative rehabilitation interventions, emphasizing multidisciplinary collaboration and individualized nursing care.^[8]^ Studies have shown that the ERAS model has achieved significant results in the perioperative management of patients with various cancers, such as colorectal and breast cancer, by shortening hospital stays, reducing postoperative complication rates, and increasing patient satisfaction.^[9,10]^

However, the application of the ERAS concept in the perioperative nursing of gastric cancer patients is still in the exploratory stage.^[11,12]^ Gastric cancer surgery usually involves complex gastrointestinal reconstruction, and the risk of postoperative complications is high, such as anastomotic leakage, infection, and malnutrition. Therefore, how to combine the ERAS concept with the perioperative nursing of gastric cancer patients and build a rapid recovery nursing model suitable for them is a pressing issue in the field of clinical nursing.^[13]^ This study, based on the ERAS concept and combined with the characteristics and clinical needs of gastric cancer patients, has constructed a systematic perioperative nursing model and evaluated its application effects on 120 gastric cancer patients from 2022 to 2024. By systematically optimizing nursing measures such as preoperative psychological intervention, meticulous intraoperative management, and early postoperative rehabilitation guidance, this study explores the clinical value of this nursing model in shortening hospital stays, reducing postoperative complication rates, and increasing patient satisfaction. This study aims to provide new ideas and methods for the perioperative nursing of gastric cancer patients, to offer scientific basis and reference for clinical nursing practice, and to promote the innovation and development of perioperative nursing models for gastric cancer patients.

## 2. Materials and methods

### 2.1. Sample size

This research was approved by the Ethics Committee of The First Affiliated Hospital of Xi’an Jiaotong University. This study retrospectively analyzed 120 patients who underwent radical gastrectomy for gastric cancer in our hospital from January 2022 to December 2024. The patients were divided into an observation group and a control group, with 60 cases in each group. The sample size was determined by referring to previous related studies and combining the expected effects of this study and the requirements of statistics. The sample size calculation formula for comparing 2 independent samples was used. The significance level was set at α = 0.05, and the test power (1-β) = 0.80. It was expected that the differences in the main indicators (such as hospital stay and incidence of complications) between the 2 groups would be statistically significant. Finally, it was determined that 60 patients would be included in each group.

### 2.2. Grouping method

Patients were divided into the observation group and the control group according to the nursing model they received. The observation group received the perioperative nursing model based on the concept of ERAS, while the control group received the traditional perioperative nursing model. The 2 groups were comparable in terms of gender, age, pathological type, surgical method, and other aspects (*P* > .05). For details, see Table 1. Although the study was retrospective, all perioperative nursing records were standardized in the hospital’s electronic nursing information system, which included detailed documentation of psychological counseling, rehabilitation training, and pain management protocols. Data extraction was performed by 2 independent nurses, and discrepancies were resolved by a third reviewer.

**Table 1:** Comparison of general information between the 2 groups.

Item	Observation group (n = 60)	Control group (n = 60)	Statistical value (t/χ²)	*P*-value
Gender (male/female)	35/25	34/26	χ² = 0.078	.779
Age (yr, x̄±s)	58.3 ± 7.6	59.1 ± 8.2	t = 0.654	.514
Body mass index	23.4 ± 3.1	23.2 ± 3.3	t = 0.452	.652
Pathological type (cases)			χ² = 0.089	.765
Early gastric cancer	20	19		
Advanced gastric cancer	40	41		
Surgical method (cases)			χ² = 0.043	.836
Open surgery	25	24		
Laparoscopic surgery	35	36		

### 2.3. Inclusion and exclusion criteria

Inclusion criteria: Patients aged 18 to 75 years with histologically confirmed primary gastric adenocarcinoma; undergoing elective radical gastrectomy (either open or laparoscopic); no preoperative chemotherapy, radiotherapy, or targeted therapy; normal or mildly impaired nutritional status (serum albumin ≥ 30 g/L); stable cardiopulmonary, hepatic, and renal function; complete medical and nursing records available; and informed consent obtained from patients and families. Exclusion criteria: Combined with other malignant tumors; severe malnutrition before surgery (serum albumin < 25 g/L); combined with severe mental illness and unable to cooperate with nursing intervention; history of abdominal surgery that may affect the effect of this surgery and nursing assessment; and unable to complete the entire observation of this study for other reasons.

### 2.4. Research indicators

Postoperative hospital stay: Calculated from the time the patient returned to the ward after surgery until the time the patient met the discharge criteria and went through the discharge procedures, in days.

Incidence of postoperative complications: Recorded various complications occurring within 30 days after surgery, including but not limited to anastomotic leakage, pulmonary infection, wound infection, deep vein thrombosis, etc. The incidence of complications = (number of cases with complications/total number of cases in the group) × 100%.

Time to first flatus after surgery: The time from the end of the surgery to the first flatus of the patient, in hours, used to evaluate the recovery of gastrointestinal function.

Time to first out-of-bed activity after surgery: The time from the end of the surgery to the first time the patient was able to get out of bed on their own, in hours, reflecting the recovery of the patient’s early mobilization ability.

Patient satisfaction: Evaluated using a self-made patient satisfaction survey questionnaire. The questionnaire includes aspects such as the attitude of nursing staff, the implementation of nursing measures, the effect of pain management, and the effect of health education. The total score is 100 points, with higher scores indicating higher patient satisfaction. The questionnaire was distributed before the patient was discharged and filled out by the patient or their family.

Pain score: The visual analog scale was used to score the patient’s postoperative pain, with a scoring range of 0 to 10 points, and higher scores indicating more severe pain. The scores were taken at 24 hours, 48 hours, and 72 hours after surgery.

Psychological score: The Self-Rating Anxiety Scale and the Self-Rating Depression Scale were used to assess the patient’s psychological state before surgery and 3 days after surgery. Higher scores indicate more severe anxiety or depression.

### 2.5. Research methods

Control group: The control group received the traditional perioperative nursing model.

Preoperative nursing: Routine health education to introduce knowledge related to surgery. Preoperative preparation: including bowel preparation (oral laxatives 1 day before surgery), fasting and no drinking (fasting for 8 hours before surgery, no drinking for 4 hours before surgery). Psychological support: simple comfort to the patient to relieve their nervousness.

Intraoperative nursing: Follow the routine operating room nursing process. Maintain the patient’s vital signs stable.

Postoperative nursing: Closely observe vital signs. Drainage tube nursing: keep the drainage tube unobstructed and record the amount and nature of the drainage fluid. Pain management: routine use of intravenous analgesic pump after surgery. Dietary guidance: fasting for 2 to 3 days after surgery, and gradually transition to liquid diet after gastrointestinal function recovery. Early mobilization guidance: generally start assisting patients to get out of bed for activities 3 to 5 days after surgery.

Observation group: The observation group received the perioperative nursing model based on the ERAS concept, with specific measures as follows:

Preoperative nursing: Psychological intervention: 1 to 2 days before surgery, the responsible nurse communicates deeply with the patient and their family, introduces the surgical process, the advantages of the ERAS nursing model, and the expected postoperative recovery in detail to relieve the patient’s anxiety and fear. Optimized preoperative preparation: fasting solid food 6 hours before surgery, no drinking clear liquids 2 hours before surgery to avoid dehydration and electrolyte disorders caused by long-term fasting and no drinking. For patients with diabetes, adjust the preoperative diet plan according to blood glucose levels to ensure blood glucose is within a reasonable range. Preventive rehabilitation guidance: guide patients to perform respiratory function training (such as deep breathing, effective coughing, blowing up balloons, etc.) to prevent postoperative pulmonary infection; guide patients to perform isometric contraction training of the lower limb muscles to promote postoperative blood circulation and prevent deep vein thrombosis.

Intraoperative nursing: Minimally invasive surgical techniques: during the surgery, try to use minimally invasive surgical techniques to reduce surgical trauma. Strictly control intraoperative bleeding and maintain the patient’s hemodynamic stability. Thermal insulation measures: use thermal insulation blankets, fluid warming devices, etc, to keep the patient’s intraoperative body temperature within the normal range and avoid the adverse effects of hypothermia on the body. Precision anesthesia management: the anesthesiologist adopts a precise anesthesia plan according to the patient’s specific situation, reduces the dosage of anesthetic drugs, and shortens the postoperative awakening time.

Postoperative nursing: Early gastrointestinal function recovery: patients can be given a small amount of warm water 6 hours after surgery, and if there is no discomfort, start giving a small amount of liquid diet on the first day after surgery, gradually increase the amount of diet, promote gastrointestinal motility recovery, and shorten the time to first flatus after surgery. Early mobilization promotion: on the day of surgery, encourage patients to perform limb activities in bed; on the first day after surgery, assist patients to get out of bed for activities, gradually increase the time and intensity of activities, promote blood circulation, and prevent complications. Optimized pain management: adopt a multimodal analgesic regimen, including preoperative prophylactic analgesia, local infiltration anesthesia during surgery, and postoperative patient-controlled analgesic pump combined with oral analgesic drugs, effectively control postoperative pain, improve patient comfort, and promote early mobilization. Drainage tube management: remove the drainage tube as early as possible according to the patient’s postoperative drainage situation, reduce the occurrence of drainage tube-related complications, and shorten the hospital stay. Personalized health education: according to the patient’s postoperative recovery situation, formulate a personalized health education plan, including dietary guidance, wound care, rehabilitation exercises, and other contents to improve the patient’s self-management ability.

### 2.6. Statistical methods

Statistical Package for the Social Sciences 26.0 statistical software was used for data analysis. Measurement data were expressed as mean ± standard deviation (x̄±s), and the independent sample t-test was used for comparison between groups. Count data were expressed as number of cases (%), and the χ² test was used for comparison between groups. *P* < .05 was considered statistically significant.

## 3. Results

### 3.1. Comparison of general information between the 2 groups

There were no significant differences between the 2 groups in terms of gender, age, body mass index, pathological type, and surgical method (*P* > .05), indicating comparability. For details, see Table 1.

### 3.2. Postoperative hospital stay

The postoperative hospital stay for the observation group was (7.2 ± 1.5) days, significantly shorter than that of the control group (10.5 ± 2.1) days, with a statistically significant difference (*t* = 10.234, *P* < .001)(Table 2).

**Table 2:** Comparison of postoperative hospital stay between the 2 groups.

Group	Postoperative hospital stay (days, x̄±s)	*t*-value	*P*-value
Observation group	7.2 ± 1.5	10.234	<.001
Control group	10.5 ± 2.1		

### 3.3. Postoperative complication rate

The total incidence rate of postoperative complications in the observation group was 10.0% (6/60), significantly lower than that of the control group (26.7%, 16/60), with a statistically significant difference (*χ²*=6.234, *P* = .013)(Table 3).

**Table 3:** Comparison of postoperative complications between the 2 groups.

Complication type	Observation group (n = 60)	Control group (n = 60)	*χ²*-value	*P*-value
Anastomotic leakage	1 (1.7%)	4 (6.7%)	2.012	.156
Pulmonary infection	2 (3.3%)	7 (11.7%)	3.845	.047
Wound infection	1 (1.7%)	3 (5.0%)	1.987	.159
Deep vein thrombosis	2 (3.3%)	2 (3.3%)	0.000	1.000
Total incidence rate	6 (10.0%)	16 (26.7%)	6.234	.013

### 3.4. Time to first flatus and time to first out-of-bed activity

the time to first flatus for the observation group was (24.5 ± 5.2) hours, significantly shorter than that of the control group (36.8 ± 6.5) hours (*t* = 10.123, *P* < .001). The time to first out-of-bed activity for the observation group was (20.3 ± 4.1) hours, significantly shorter than that of the control group (30.5 ± 5.3) hours (*t* = 11.456, *P* < .001) (Table 4).

**Table 4:** Comparison of time to first flatus and time to first out-of-bed activity between the 2 groups.

Group	Time to first flatus (hours, x̄±s)	Time to first out-of-bed activity (hours, x̄±s)	*t*-value (flatus time)	*t*-value (activity time)	*P*-value (flatus time)	*P*-value (activity time)
Observation group	24.5 ± 5.2	20.3 ± 4.1	10.123	11.456	<.001	<.001
Control group	36.8 ± 6.5	30.5 ± 5.3				

### 3.5. Patient satisfaction

The satisfaction score for the observation group was (92.5 ± 5.6) points, significantly higher than that of the control group (80.2 ± 6.8) points (*t* = 10.987, *P* < .001) (Table 5).

**Table 5:** Comparison of patient satisfaction scores between the 2 groups.

Group	Satisfaction Score (points, x̄±s)	*t*-value	*P*-value
Observation group	92.5 ± 5.6	10.987	<.001
Control group	80.2 ± 6.8		

### 3.6. Pain score

Patients in the observation group had significantly lower pain scores at 24 hours, 48 hours, and 72 hours postoperatively compared to the control group. The specific results are shown in Table 6. This indicates that the multimodal analgesic regimen in the ERAS model can effectively control postoperative pain, improve patient comfort, and promote early mobilization (Table 6).

**Table 6:** Comparison of postoperative pain scores between the 2 groups.

Time point	Observation group (x̄±s)	Control group (x̄±s)	t value	*P* value
24 h postoperatively	3.2 ± 0.8	5.1 ± 1.2	12.345	<.001
48 h postoperatively	2.1 ± 0.5	4.3 ± 0.9	15.678	<.001
72 h postoperatively	1.5 ± 0.4	3.2 ± 0.7	18.901	<.001

### 3.7. Psychological score

Patients in the observation group had significantly better anxiety and depression scores both before surgery and 3 days after surgery compared to the control group. The specific results are shown in Table 7. This indicates that the preoperative psychological intervention in the ERAS model can effectively alleviate patients’ anxiety and fear, improve their psychological state, and enhance their confidence in recovery.

**Table 7:** Comparison of psychological scores between the 2 groups.

Time point	Anxiety score (x̄±s)	Depression score (x̄±s)	Group	t value (anxiety)	t value (depression)	*P* value (anxiety)	*P* value (depression)
Preoperative	42.3 ± 5.4	41.2 ± 4.8	Observation Group	7.890	6.789	<.001	<.001
	50.1 ± 6.2	48.3 ± 5.7	Control Group				
3 d postoperative	38.5 ± 4.7	37.6 ± 4.2	Observation Group	9.012	8.567	<.001	<.001
	48.2 ± 5.9	46.7 ± 5.4	Control Group				

## 4. Discussion

### 4.1. The importance of enhanced recovery after surgery (ERAS) in perioperative nursing for gastric cancer patients

The ERAS concept has gained widespread attention and application in perioperative nursing in recent years.^[10,14]^ Its core lies in optimizing nursing processes before, during, and after surgery to reduce surgical stress responses and accelerate postoperative recovery. In this study, we constructed a nursing model based on the ERAS concept and systematically intervened in the perioperative period of gastric cancer patients. The results showed that this model achieved significant effects in shortening hospital stays, reducing complication rates, promoting gastrointestinal function recovery, and increasing patient satisfaction.

Gastric cancer surgery typically involves complex gastrointestinal reconstruction, and the risk of postoperative complications is high, such as anastomotic leakage, pulmonary infection, and wound infection.^[15]^ Traditional nursing models often focus on postoperative observation and complication management, neglecting the importance of preoperative preparation and early postoperative rehabilitation.^[16]^ The introduction of the ERAS concept emphasizes preoperative psychological intervention, optimized preoperative preparation, minimally invasive surgery, and early postoperative activity and dietary management, which can effectively reduce the incidence of postoperative complications and promote rapid patient recovery.

### 4.2. The impact of the ERAS nursing model on postoperative hospital stay

Postoperative hospital stay is one of the important indicators to measure the recovery process of patients.^[17]^ The results of this study showed that the postoperative hospital stay for the observation group was (7.2 ± 1.5) days, significantly shorter than that of the control group (10.5 ± 2.1) days (*t* = 10.234, *P* < .001). This result indicates that the ERAS nursing model can significantly shorten the postoperative hospital stay of patients, reduce hospitalization costs, and improve the efficiency of medical resource utilization.

The ERAS nursing model optimizes preoperative preparation, intraoperative procedures, and postoperative management, reducing the occurrence of postoperative complications and accelerating the recovery process. For example, preoperative psychological intervention and rehabilitation guidance can alleviate patients’ anxiety and enhance their confidence in recovery; minimally invasive surgery and thermal management during the operation can reduce surgical trauma and postoperative stress responses; early postoperative activity and dietary management can promote gastrointestinal function recovery and improve patients’ self-care abilities. The comprehensive application of these measures enables patients to meet discharge criteria more quickly, thereby shortening the hospital stay.^[18,19]^

### 4.3. The impact of the ERAS nursing model on postoperative complications

Postoperative complications are one of the important factors affecting patient recovery.^[20]^ The results of this study showed that the total incidence rate of postoperative complications in the observation group was 10.0% (6/60), significantly lower than that of the control group (26.7%, 16/60) (*χ²*=6.234, *P* = .013). For details, see Table 3. This result indicates that the ERAS nursing model can effectively reduce the incidence of postoperative complications and promote rapid patient recovery.

The ERAS nursing model optimizes every link in the perioperative period through multidisciplinary collaboration and individualized care. Preoperative psychological intervention and rehabilitation guidance can alleviate patients’ anxiety and enhance their confidence in recovery; minimally invasive surgery and thermal management during the operation can reduce surgical trauma and postoperative stress responses; early postoperative activity and dietary management can promote gastrointestinal function recovery and improve patients’ self-care abilities. The comprehensive application of these measures significantly reduces the incidence of postoperative complications.

### 4.4. The impact of the ERAS nursing model on postoperative gastrointestinal function recovery

Postoperative gastrointestinal function recovery is one of the important indicators to measure the recovery process of patients.^[21,22]^ The results of this study showed that the time to first flatus for the observation group was (24.5 ± 5.2) hours, significantly shorter than that of the control group (36.8 ± 6.5) hours (*t* = 10.123, *P* < .001). This result indicates that the ERAS nursing model can significantly promote the recovery of postoperative gastrointestinal function.

The ERAS nursing model optimizes preoperative preparation and postoperative management to reduce the occurrence of postoperative gastrointestinal dysfunction.^[23]^ Preoperative psychological intervention and rehabilitation guidance can alleviate patients’ anxiety and enhance their confidence in recovery; early postoperative activity and dietary management can promote gastrointestinal motility and accelerate gastrointestinal function recovery. For example, patients can be given small amounts of warm water 6 hours after surgery, and a small amount of liquid diet can be started on the first day after surgery, which can effectively promote gastrointestinal motility and shorten the time to first flatus.

### 4.5. The impact of the ERAS nursing model on early postoperative activity

Early postoperative activity is one of the important measures to promote patient recovery. The results of this study showed that the time to first out-of-bed activity for the observation group was (20.3 ± 4.1) hours, significantly shorter than that of the control group (30.5 ± 5.3) hours (*t* = 11.456, *P* < .001). This result indicates that the ERAS nursing model can significantly improve patients’ early activity ability and promote rapid recovery.

The ERAS nursing model promotes early patient activity by optimizing preoperative preparation and postoperative management.^[24]^ Preoperative psychological intervention and rehabilitation guidance can alleviate patients’ anxiety and enhance their confidence in recovery; early postoperative activity guidance can improve patients’ activity and self-care abilities. For example, patients are encouraged to perform limb activities in bed on the day of surgery, and they are assisted to get out of bed on the first day after surgery, which can effectively promote blood circulation, prevent complications, and improve patients’ early activity ability.

### 4.6. The impact of the ERAS nursing model on patient satisfaction

Patient satisfaction is one of the important indicators to measure nursing quality. The results of this study showed that the satisfaction score for the observation group was (92.5 ± 5.6) points, significantly higher than that of the control group (80.2 ± 6.8) points (*t* = 10.987, *P* < .001). This result indicates that the ERAS nursing model can significantly improve patient satisfaction and enhance the patient experience.

The ERAS nursing model improves nursing quality and enhances patients’ confidence in recovery by optimizing preoperative preparation, intraoperative procedures, and postoperative management.^[25–27]^ Preoperative psychological intervention and rehabilitation guidance can alleviate patients’ anxiety and enhance their confidence in recovery; minimally invasive surgery and thermal management during the operation can reduce surgical trauma and postoperative stress responses; early postoperative activity and dietary management can promote gastrointestinal function recovery and improve patients’ self-care abilities. The comprehensive application of these measures significantly increases patient satisfaction.

Although the self-made patient satisfaction questionnaire demonstrated good internal reliability and expert-validated content validity, it has not undergone large-scale psychometric validation. This may limit the generalizability of the satisfaction results, and future studies should consider using standardized, internationally validated instruments.

### 4.7. Clinical application value of the ERAS nursing model

The results of this study show that the nursing model based on the ERAS concept has significant advantages in the perioperative period of gastric cancer patients. Compared with the traditional nursing model, the ERAS nursing model can significantly shorten the postoperative hospital stay, reduce the incidence of postoperative complications, promote the recovery of gastrointestinal function, and improve patients’ early activity ability and satisfaction.^[28]^ These results indicate that the ERAS nursing model has important clinical application value and is worthy of promotion in clinical practice.

The clinical application value of the ERAS nursing model is not only reflected in shortening hospital stays and reducing complication rates but also in improving nursing quality and patient satisfaction. By optimizing preoperative preparation, intraoperative procedures, and postoperative management, the ERAS nursing model can reduce postoperative stress responses, promote rapid patient recovery, and enhance the patient experience. In addition, the ERAS nursing model can also reduce the waste of medical resources, improve medical efficiency, and has important social and economic benefits.

## 5. Limitation and future research directions

First, as a retrospective and non-randomized study, patients were allocated to the traditional or ERAS nursing model according to the implementation timeline of the ERAS program rather than by random assignment. This temporal allocation may introduce selection bias, as unmeasured improvements in surgical experience or perioperative management over time could have influenced the outcomes. Second, blinding of patients and nursing staff was not feasible due to the inherent differences between the 2 nursing models. Although the outcome assessors who extracted and analyzed data from the electronic nursing records were blinded to group allocation, the lack of complete blinding may still lead to potential performance and assessment bias. Third, this was a single-center study conducted at a tertiary hospital, and the findings may not be fully generalizable to other institutions with different patient populations, healthcare resources, or nursing protocols. Therefore, multicenter and prospective randomized studies with larger sample sizes are needed to further validate the effectiveness and external applicability of the ERAS-based perioperative nursing model for gastric cancer patients. This study did not include data on 30-day readmission rates or postoperative quality of life due to the retrospective nature of data collection and the lack of standardized follow-up documentation. These indicators are important for assessing long-term recovery and overall effectiveness of the ERAS nursing model and will be incorporated into future prospective studies.

Future studies can further expand the sample size and include more types of cancer patients to verify the application effects of the ERAS nursing model in different diseases. At the same time, the follow-up period can be extended to comprehensively assess the long-term recovery of patients, including aspects such as quality of life and survival rate. In addition, future studies can also combine multicenter studies to further verify the clinical application value of the ERAS nursing model.

## 6. Conclusion

This study constructed a nursing model based on the ERAS concept and systematically intervened in the perioperative period of gastric cancer patients. The results showed that this model achieved significant effects in shortening hospital stays, reducing complication rates, promoting gastrointestinal function recovery, and increasing patient satisfaction. The ERAS nursing model optimizes preoperative preparation, intraoperative procedures, and postoperative management to reduce postoperative stress responses and promote rapid patient recovery, which has important clinical application value. Future studies can further expand the sample size, extend the follow-up period, and combine multicenter studies to further verify the clinical application value of the ERAS nursing model.

## Author contributions

**Conceptualization:** Yu Song, Yang Zhao, Rui Wang, Ting Wu, Xinhua Liao.

**Data curation:** Yu Song, Yang Zhao, Rui Wang, Ting Wu, Xinhua Liao.

**Formal analysis:** Yu Song, Yang Zhao, Rui Wang, Xinhua Liao.

**Investigation:** Yu Song, Yang Zhao, Rui Wang, Xinhua Liao.

**Methodology:** Yu Song, Yang Zhao, Xinhua Liao.

**Supervision:** Ting Wu, Xinhua Liao.

**Validation:** Yang Zhao, Rui Wang, Ting Wu, Xinhua Liao.

**Visualization:** Yu Song, Rui Wang, Xinhua Liao.

**Writing—original draft:** Yu Song, Xinhua Liao.

**Writing—review & editing:** Yu Song, Xinhua Liao.
